# Non-Eosinophilic Nasal Polyps Shows Increased Epithelial Proliferation and Localized Disease Pattern in the Early Stage

**DOI:** 10.1371/journal.pone.0139945

**Published:** 2015-10-06

**Authors:** Dong-Kyu Kim, Hong Ryul Jin, Kyoung Mi Eun, Somasundran Mutusamy, Seong H. Cho, Sohee Oh, Dae Woo Kim

**Affiliations:** 1 Department of Otorhinolaryngology-Head and Neck Surgery, Chuncheon Sacred Heart Hospital and Nano-Bio Regenerative Medical Institute, Hallym University College of Medicine, Chuncheon, Republic of Korea; 2 Department of Otorhinolaryngology-Head and Neck Surgery, Boramae Medical Center, Seoul National University College of Medicine, Seoul, Korea; 3 Division of Allergy-Immunology, Department of Internal Medicine, University of South Florida Morsani College of Medicine, Tampa, Florida; 4 Department of Biostatistics, Boramae Medical Center, Seoul National University College of Medicine, Seoul, Korea; University Medical Center of Princeton/Rutgers Robert Wood Johnson Medical School, UNITED STATES

## Abstract

**Background:**

Non-eosinophilic nasal polyps (NPs) show less inflammatory changes and are less commonly associated with lower airway inflammatory disorders such as asthma, compared with eosinophilic NPs. However, the development of non-eosinophilic NPs which is a predominant subtype in Asian population still remains unclear.

**Methods:**

A total of 81 patients (45 with non-eosinophilic NPs and 36 with eosinophilic NPs) were enrolled. Clinical information and computed tomography (CT), endoscopic, and histological findings were investigated. Tissue samples were analyzed for total IgE levels and for mRNA expression levels of interleukin (IL)-4, IL–5, IL–13, interferon (IFN)-γ, tumor necrosis factor (TNF)-α, IL-17A, IL–22, IL-23p19, transforming growth factor (TGF)-β1, TGF-β2, TGF-β3, and periostin. Immunostaining assessment of Ki–67 as a proliferation marker was performed.

**Results:**

We found that epithelial in-growing patterns such as pseudocysts were more frequently observed in histological and endoscopic evaluations of non-eosinophilic NPs, which was linked to increase epithelial staining of Ki–67, a proliferating marker. Eosinophilic NPs were characterized by high infiltration of inflammatory cells, compared with non-eosinophilic NPs. To investigate the developmental course of each subtype, CT was analyzed according to CT scores and subtypes. Non-eosinophilic NPs showed more localized pattern and maxillary sinus involvement, but lesser olfactory involvement in early stage whereas eosinophilic NPs were characterized by diffuse ethmoidal and olfactory involvement. In addition, high ethmoidal/maxillary (E/M) CT scores, indicating ethmoidal dominant involvement, were one of surrogate markers for eosinophilic NP. E/M CT scores was positively correlated with levels of T_H_2 inflammatory markers, including IL–4, IL–5, periostin mRNA expression and total IgE levels in NPs, whereas levels of the T_H_1 cytokine, IFN- γ were inversely correlated. Moreover, if the combinatorial algorithm meet the three of the four markers, including IL–5 (<2.379), periostin (<3.889), IFN-γ (>0.316), and E/M ratio (<2.167), non-eosinophilic CRSwNP are diagnosed with a sensitivity of 84.4% and a specificity of 84.8%.

**Conclusion:**

Histologic, immunologic and clinical data suggest that non-eosinophilic NPs showed enhanced epithelial alteration and more localized maxillary involvement. Combination of cutoff value on IL–5, periostin, IFN-γ, and E/M scores may be one of surrogate markers for non-eosinophil NP subtype.

## Introduction

Chronic rhinosinusitis (CRS) is one of the most common chronic rhinologic diseases and can significantly reduce the quality of life of affected persons. CRS is characterized by accumulation of inflammatory cells and marked tissue remodeling, and is considered a multifactorial disease within a heterogeneous group [1–3]. This disease is usually classified into one of two phenotypes, CRS with nasal polyps (CRSwNP) and CRS without nasal polyps, based primarily on endoscopic findings [2]. However, clinical phenotypes do not provide full insight between races, because patients with CRSwNP show different immunohistologic features in Western and Asian countries. Growing evidence suggests that CRSwNP in Western patients is characterized by a T_H_2-based immune response with high interleukin (IL)-5 levels and abundant eosinophilic infiltration, whereas studies of CRSwNP in Asian patients predominantly show a mixed T cell immune response and non-eosinophilic inflammation [3–9].

Due to this heterogeneity, CRSwNP patients were distinguished two subtypes such as eosinophilic and non-eosinophilic NPs [10]. In patients with CRSwNP, patients show different levels of inflammatory cell accumulation and remodeling patterns were different according to the subtypes [10–16]. For example, in eosinophilic NPs, eosinophils may contribute to edema, whereas neutrophilic infiltration has an important role in vigorous glandular hypertrophy and subsequent fibrosis rather than edema in non-eosinophilic NPs. CRSwNP subtype is also important for the development of an appropriate personalized treatment plan, because CRSwNP may have clinical features that differ between eosinophilic and non-eosinophilic NPs. These features include characteristic symptoms, severity of disease, co-morbidity, clinical course after surgery and response of anti-IgE or anti-IL–5 [9, 17, 18]. Non-eosinophilic CRSwNP is regarded as an extrinsic rhinosinusitis, because the inflammation originates from external stimuli such as bacteria and allergens rather than the intrinsic mucosal abnormalities [19]. Moreover, US 2^nd^ generation nasal polyps defined as subjects with self-reported Asian ancestry and born in the United States, showed similar clinical patterns to native-born Asian patients, which is suggestive of possible genetic factors contributing to the pathogenesis of non-eosinophilic NPs [20]. To date, although there are some suggestions on development of non-eosinophilic NPs, few studies investigating the differences of histological, clinical and molecular parameters among patients with CRSwNP in Asian population, has been reported.

## Materials and Methods

### Subjects

We retrospectively studied patients who were diagnosed as CRSwNP. NP tissues were obtained from routine, functional, endoscopic sinus surgery in patients with CRSwNP [2]. The diagnosis of CRSwNP was based on personal history, physical examination, nasal endoscopy, and CT findings on the sinuses according to the EPOS (European position paper on rhinosinusitis and nasal polyps) 2012 guidelines. All patients provided written informed consent, and this study was approved by the institutional review board of Seoul National University Hospital and Boramae Medical Center. Three exclusion criteria were employed patients younger than 18 years of age; patients previously treated with antibiotics, systemic or topical corticosteroids, or other immune-modulating drugs for 4 weeks; and patients with antrochoanal polyps, allergic fungal sinusitis, cystic fibrosis, or immotile ciliary disease. In this study, each NP tissue sample was divided into three parts: One third was fixed in 10% formaldehyde and embedded in paraffin for histological analysis, another third was immediately frozen and stored at -80°C for subsequent isolation of mRNA, and the final third of the tissue was submersed in 1mL phosphate-buffered saline supplemented with 0.05% Tween–20 (Sigma-Aldrich, St Louis, MO, USA) and 1% protease inhibitor cocktail (Sigma-Aldrich) per 0.1 g tissue. This tissue was homogenized with a mechanical homogenizer at 1,000 rpm for 5 min on ice. After homogenization, the suspensions were centrifuged at 3,000 rpm for 10 min at 4°C. The supernatants were separated and stored at -80°C for further analysis of total IgE. The atopic status of study patients was evaluated using the ImmunoCAP^®^ assay (Phadia, Uppsala, Sweden), which detects IgE antibodies against six mixtures of common aeroallergens (house dust mites, molds, trees, weeds, grass, and animal dander). Patients were considered atopic if the allergen-specific IgE level was greater than 3.51 kU/L [21]. A diagnosis of asthma was performed by an allergist based on medical history and lung function analysis, including challenge tests. The Lund-MacKay scoring system (0–24) was used to grade the severity of sinus disease. In addition to the Lund-MacKay scoring system [22], we used the difference between the higher-and lower-side CT scores as a metric for the degree of disease localization, the opacification of the entire olfactory cleft on CT images (0, no opacification; 1, <25% opacification; 2, 25–50% opacification; 3, 50–75% opacification; 4, >75% opacification) [23], and the ratio of the ethmoid and maxillary sinus scores (E/M ratio) based on Lund-MacKay scoring. If the E/M ratio was greater than 2, opacification of the ethmoid sinus was considered to be more severe than that of the maxillary sinus. By histological analysis, we assessed the proportion of observed NPs occupied by a pseudocyst using a grading system: 0, none; 1, less than 25% of tissue area; 2, 25–50% of tissue area; 3, 50–75% of tissue area; and 4, greater than 75% of tissue area.

### Definition of polyps: eosinophilic vs. non-eosinophilic

The paraffin tissue sections (5 μm) were stained with hematoxylin and eosin. The stained sections were evaluated by two independent physicians who were blinded to the respective diagnosis and clinical data. The number of eosinophils was counted in a high-power field (400×), in which eosinophils in the mucosa were the densest cellular infiltrate beneath the epithelium. Five visual fields were examined per section to determine the mean percentage of eosinophils out of the total number of inflammatory cells. Patients with CRSwNP were classified as eosinophilic CRSwNP when more than 10% of the total observed infiammatory cells were eosinophils and as non-eosinophilic CRSwNP otherwise [24].

### Immunohistochemistry

Immunohistochemistry was performed with the Polink–1 horseradish peroxidase (HRP) Broad Spectrum DAB Detection Kit (Golden Bridge International Labs., WA, USA). Briefly, after deparaffinization, the sections were incubated in 3% hydrogen peroxidase for endogenous peroxidase inhibition and then heated in a microwave in 10 mM citrate buffer (pH 6.0) for heat-induced epitope retrieval. The sections were incubated for 60min at room temperature with each primary antibody. The primary antibodies were: mouse anti-human eosinophil major basic protein (EMBP; 1:100; Merck Millipore, Darmstadt, Germany), mouse anti-human neutrophil elastase (1:100; Abcam, Cambridge, UK), and rabbit anti-human Ki–67 (1:1; Abcam). A proteinase treatment with 0.1% trypsin for 15 minutes in water was performed prior to blocking and antibody staining, particularly during EMBP staining. The sections were incubated in broad antibody enhancer and polymer-HRP for rabbit and mouse antibodies and then stained with the DAB Detection System. Finally, slides were counterstained with hematoxylin. The positive cells in epithelia, glands, and submucosa were counted in the densest five visual fields (400×) by two independent observers, and the average of the resulting scores was used.

### Quantitative real-time PCR

The mRNA levels of IL–4, IL–5, IL–13, interferon (IFN)-γ, tumor necrosis factor (TNF)-α, IL-17A, IL–22, IL-23p19, transforming growth factor (TGF)-β1, TGF-β2, TGF-β3, and periostin in NP tissues were evaluated using quantitative, real-time reverse transcription-PCR (qRT-PCR) analysis as previously described (23). Briefly, total RNA was extracted from tissue samples with TRIzol reagent (Invitrogen, Carlsbad, CA, USA). One microgram of total RNA was then reverse-transcribed into cDNA using a cDNA Synthesis Kit (amfiRivert Platinum cDNA Synthesis Master Mix, GenDEPOT, Barker, TX, USA). qRT-PCR was performed with Light Cycler 480 SYBR Green I Master mix (Roche, Mannheim, Germany) using specific primers. The primer sequences were as follows: GAPDH, 5´-CATGGGTGTGAACCATGAGAA–3´ for the forward primer and 5´-GGTCATGAGTCCTTCCACGAT–3´ for the reverse primer; TGF-β1, 5´-TGAACCGGCCTTTCCTGCTTCTCATG–3´ for the forward primer and 5´-GCGGAAGTCAATGTACAGCTGCCGC–3´ for the reverse primer; TGF-β2, 5´-TGGATGCGGCCTATTGCTTTA–3´ for the forward primer and 5´- GCGGAAGTCAATGTACAGCTGCCGC–3´ for the reverse primer; and TGF-β3, 5´-GTGAGTGGCTGTTGAGAAGAGA–3´ for the forward primer and 5´-GAGGATTAGAGGGTTGTGG–3´ for the reverse primer. In addition, for analysis of IL–4 (Hs00174122_m1), IL–5 (Hs01548712_g1), IL–13 (Hs00174379_m1), IFN-γ (Hs00989291_m1), TNF-α (Hs01113624_g1), IL-17A (Hs00174383_m1), IL–22 (Hs01574154_m1), IL-23p19 (Hs00900828_g1), and periostin (Hs01566734_m1), pre-developed assay reagent kits of primers and probes were purchased for TaqMan assays (Life Technologies Korea, Seoul, Korea). Cycling conditions were 95°C for 5 min, followed by 60 cycles at 95°C for 15 sec, 55°C for 20 sec, and 72°C for 20 sec. For normalization, we measured the expression of the GAPDH housekeeping gene. Data were analyzed with Sequence Detection Software version 1.9.1 (Applied Biosystems, Foster City, CA, USA). Relative gene expression was calculated using the comparative 2^-ΔΔCT^ method.

### Statistical analysis

Statistical analyses were performed using IBM SPSS 20 (SPSS, Inc., Chicago, IL, USA) and GraphPad Prism 6.0 (GraphPad Software Inc., La Jolla, CA, USA). In this study, Mann-Whitney *U*-test for unpaired comparison was used. Test for normality was confirmed by Kolmogorov-Smirnov test. The Fisher's exact test was also used to determine whether there were associations between endotype of CRSwNP and pseudocyst. The Spearman correlation coefficient was utilized to determine variable relationship because the data were not normally distributed such as ordinary and continuous variables. Finally, receiver-operating characteristic (ROC) curve analysis was used to identify the surrogate marker cutoff values for prediction of non-eosinophilic CRSwNP. The box-and-whisker plot represents the median and the lower to upper quartile as the box form, and the 5 to 95 percentile as the whiskers. The significance level was set at α = 0.05 (*P<.05, **P<.01, and ***P<.001).

## Results

The study population in this work included 81 CRSwNP subjects. Among these, 45 (55.6%) were classified as non-eosinophilic CRSwNP, and the rest (n = 36, 44.4%) were classified as eosinophilic CRSwNP. A lower incidence of atopy and blood eosinophilia was observed in non-eosinophilic CRSwNP compared with eosinophilic CRSwNP (Table 1).

**Table 1:** Patient characteristics and methods used.

Patient characteristics	Non-eosinophilic nasal polyps (n = 45)	Eosinophilic nasal polyps (n = 36)	P value
Mean age, years, mean (SD)	49 (15)	48 (15)	0.331
Atopy, n (%)	11 (24%)	17 (47%)	0.019
Asthma, n (%)	3 (6.6%)	5 (13.9%)	0.279
Lund-Mackay CT score, median (IQR)	15 (10–20)	16 (10–22)	0.330
Blood eosinophils,/mm^3^ (SD)	121.9 (120.7)	156.1 (112.7)	0.014
**Methodologies used, n**			
CT	43	36	N/A
Endoscopic photo analysis	22	23	N/A
Histologic evaluation	22	23	N/A
Tissue mRNA	36	26	N/A
Tissue homogenate	15	14	N/A

N/A: Not applicable

### Increased epithelial alteration in non-eosinophilic CRSwNP

**Fig 1:**
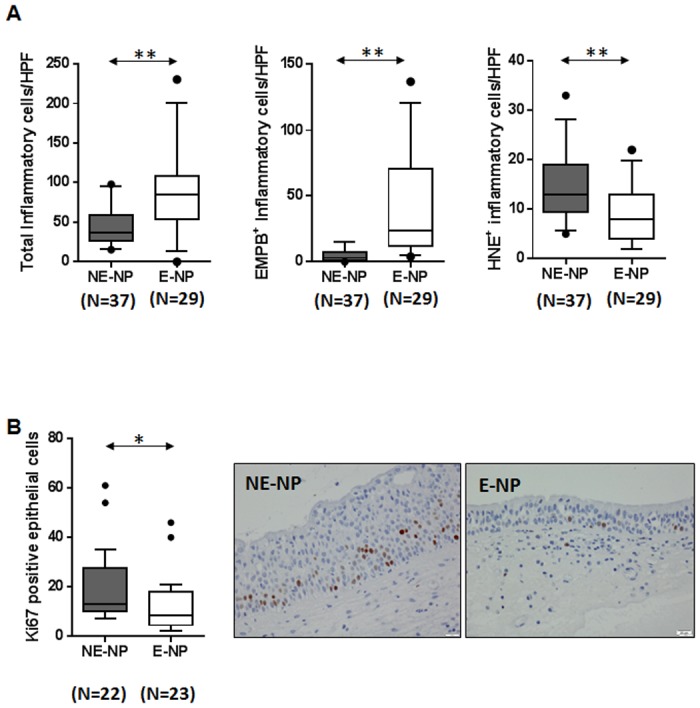
**(A)** Comparison of total inflammatory cells, EMBP^+^ cells, and HNE^+^ cell were performed in NP tissues between non-eosinophilic and eosinophilic CRSwNP patients **(B)** Expression of Ki–67 in NP tissues from non-eosinophilic and eosinophilic CRSwNP patients. Number of Ki-67-positive epithelial cells per 100 cells, averaged from three different areas of epithelium (*p<0.05, **p<0.01, and ***p<0.001). Data represent means (SD). E-NP, eosinophilic nasal polyp; NE-NP, non-eosinophilic nasal polyp.

To investigate the distribution of inflammatory cells, we performed immunohistochemistry on NPs (N = 66, Fig 1A). Examination of the total inflammatory cells population in NPs showed that greater numbers of those were present in eosinophilic CRSwNP. In addition, eosinophilic CRSwNP showed a significantly greater number of EMBP^+^ cells compared to non-eosinophilic CRSwNP. In contrast, non-eosinophilic CRSwNP had increased numbers of HNE^+^ cells, compared to the eosinophilic CRSwNP. If non-eosinophilic, or “extrinsic,” CRSwNP is primarily affected by external stimuli, the appearance of epithelial proliferating markers would be more pronounced than in eosinophilic CRSwNP. To verify this hypothesis, we performed an immunohistochemical analysis using Ki–67. The number of cells observed to be Ki–67 positive, which indicates proliferating cells, was significantly greater in the epithelial layer from patients with non-eosinophilic CRSwNP compared with those with eosinophilic CRSwNP (Fig 1B). Because we identified increased epithelial proliferation in non-eosinophilic CRSwNP, we next sought to elucidate the epithelial histological difference between the two subgroups.

**Fig 2:**
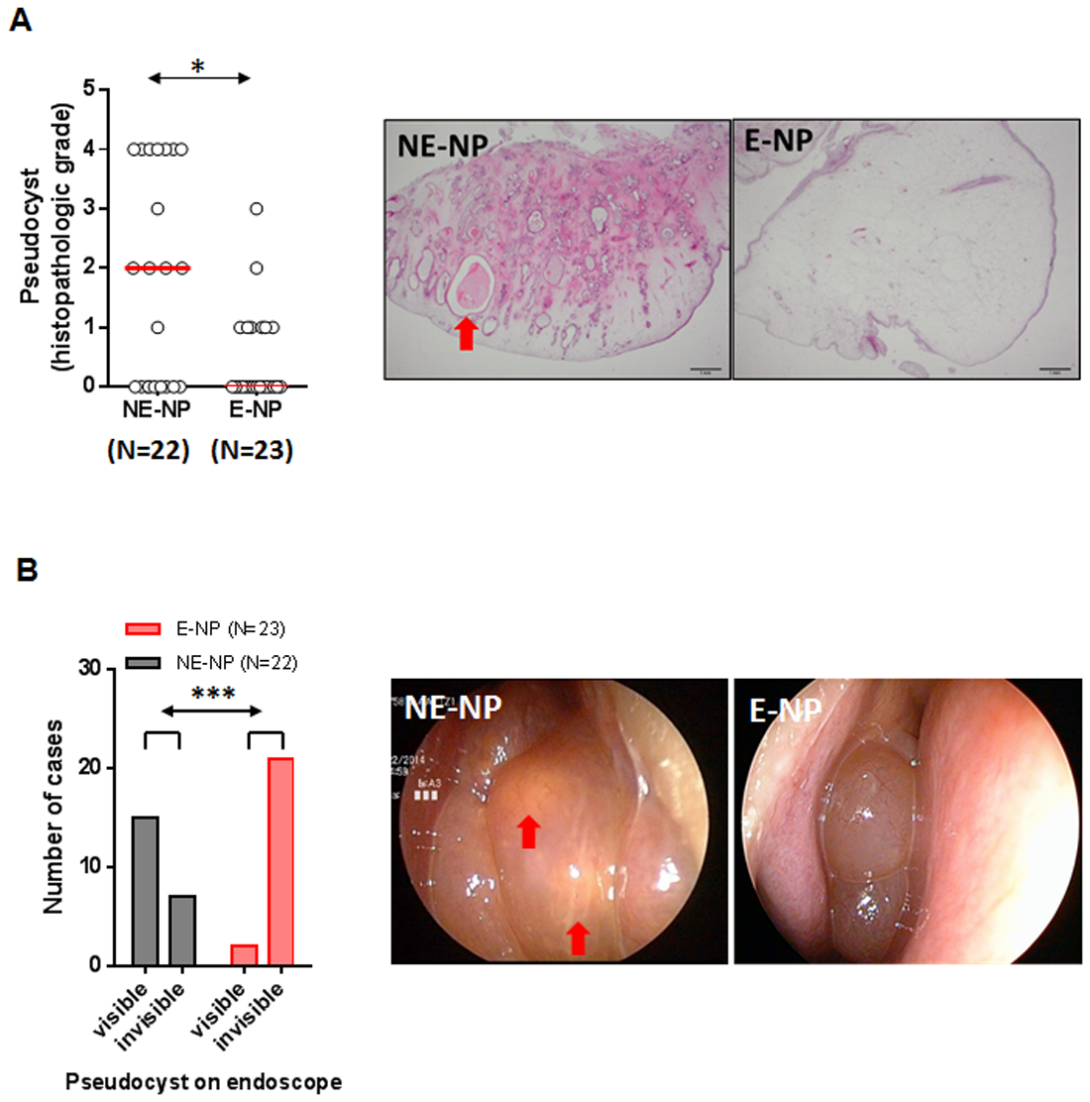
Distinctive histological features of non-eosinophilic CRSwNP patients. **(A)** Comparison of the proportion of total NP tissue area occupied by pseudocysts in different endotypes of CRSwNP and representative hematoxylin/eosin staining images of a pseudocyst (red arrow) within the epithelial layer from non-eosinophilic NP tissues (40×) **(B)** Comparison of a yellowish spot in NP tissue between non-eosinophilic and eosinophilic CRSwNP patients and representative images of a yellowish spot (red arrow) found on endoscopy in a non-eosinophilic CRSwNP patient. (*p<0.05, **p<0.01, and ***p<0.001). Data represent medians. E-NP, eosinophilic nasal polyp; NE-NP, non-eosinophilic nasal polyp.

Interestingly, pseudocysts were observed in the epithelial layer, primarily in non-eosinophilic CRSwNP. This distinctive histopathological feature of non-eosinophilic NPs was observed in a low-power field (P×) (Fig 2A). This analysis revealed that the pseudocysts occupied a significantly larger area of NPs in non-eosinophilic CRSwNP patients compared with eosinophilic CRSwNP patients (Fig 2A). Furthermore, under endoscopy, we could detect yellowish spots on NPs with the appearance of mucous retention cysts (Fig 2B). These spots were observed at a significantly higher incidence in patients with non-eosinophilic CRSwNP than in patients with eosinophilic CRSwNP (Fig 2B). Similar to the Ki–67 staining results, these epithelial alteration findings also suggest that non-eosinophilic CRSwNP arise from localized lesion, compared with eosinophilic CRSwNP.

### Differences in CT findings between subtypes of CRSwNP

**Fig 3:**
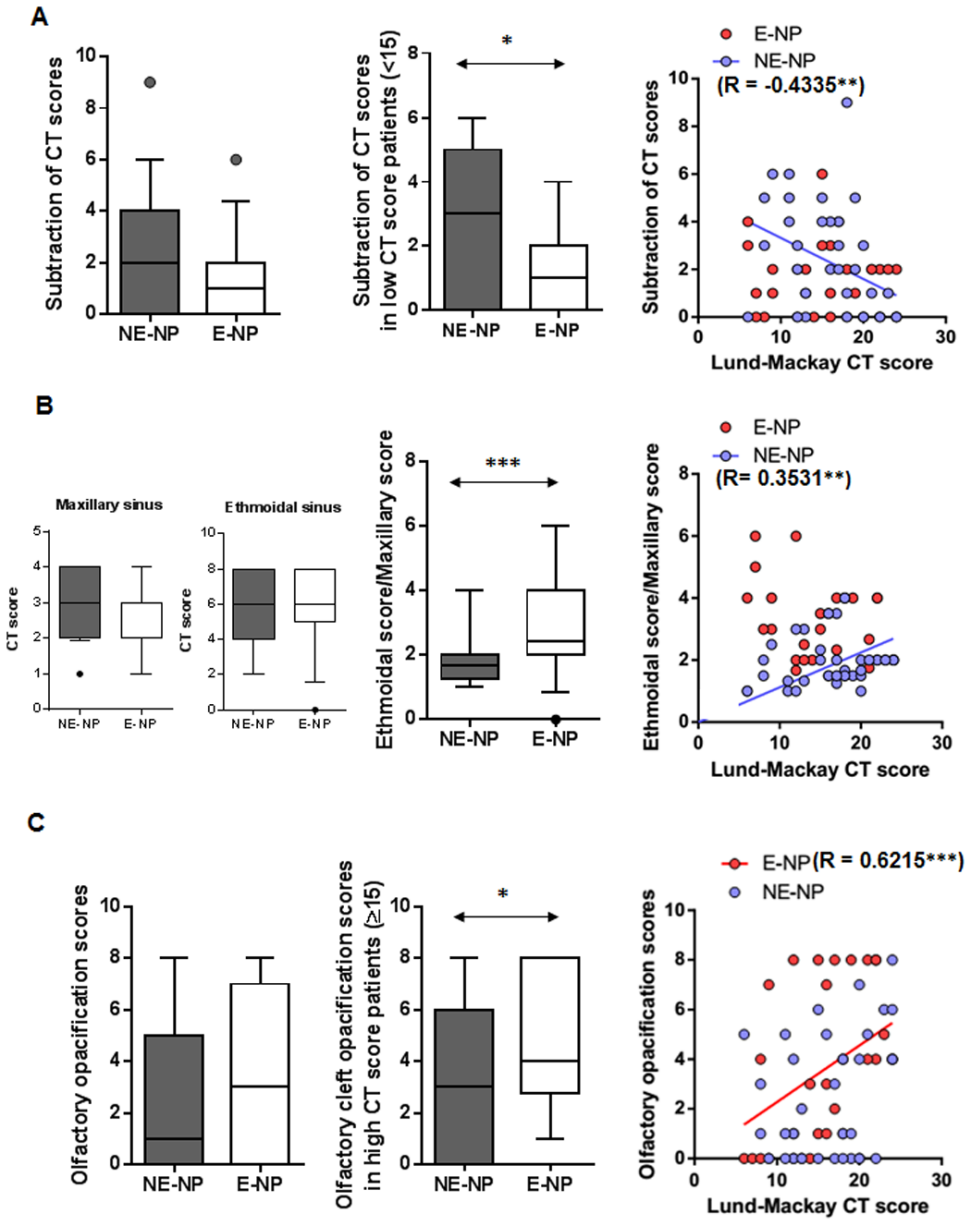
Comparison of clinical CT findings and correlation between Lund-MacKay CT score/clinical CT findings in different endotypes of CRSwNP, including (A) subtraction of CT scores, (B) olfactory opacification scores, and (C) ethmoidal scores and maxillary scores (*p<0.05, **p<0.01, and ***p<0.001). Data represent medians (IQR). E-NP, eosinophilic nasal polyp; NE-NP, non-eosinophilic nasal polyp.

Majority of nasal flow, around 70%, blows through the middle meatus in which is theoretically exposed to external stimuli [25]. If non-eosinophilic nasal polyps are mainly attributed to external stimuli, highly exposed areas like ostio-meatal unit should be involved especially in early disease status. Narrow space such as olfactory cleft, where it is difficult for external stimuli to be reached, may be less likely involved in non-eosinophilic nasal polyps. We determined whether early stages of the disease are localized or diffuse in each subtype of CRSwNP, using subtracted value of the CT scores from each side (N = 79). The subtraction of less severe side CT scores from the opposite side showed no significant differences between the two subtypes. However, when the subtracted CT scores in early stage disease (Lund-MacKay scores <15) were evaluated, we found that these subtracted values were higher in non-eosinophilic CRSwNP patients than in eosinophilic patients (Fig 3A). However, the extent of disease was negatively correlated with the CT subtraction scores in non-eosinophilic CRSwNP patients, which imply that non-eosinophilic NPs progress from unilateral or localized to bilateral or generalized diseases. In addition, ethmoidal/maxillary CT scores were significantly lower in patients with non-eosinophilic CRSwNP compared with patients with eosinophilic CRSwNP (Fig 3B). Meanwhile, eosinophilic CRSwNP patients showed a higher olfactory cleft opacification score, which correlated with CT score, compared with non-eosinophilic CRSwNP patients (Fig 3C). These findings suggest that non-eosinophilic NPs start locally, especially in the OMU area where is susceptible to external stimuli, leading to maxillary involvement.

### Relationship between parameters of CT images and immunologic features

**Fig 4:**
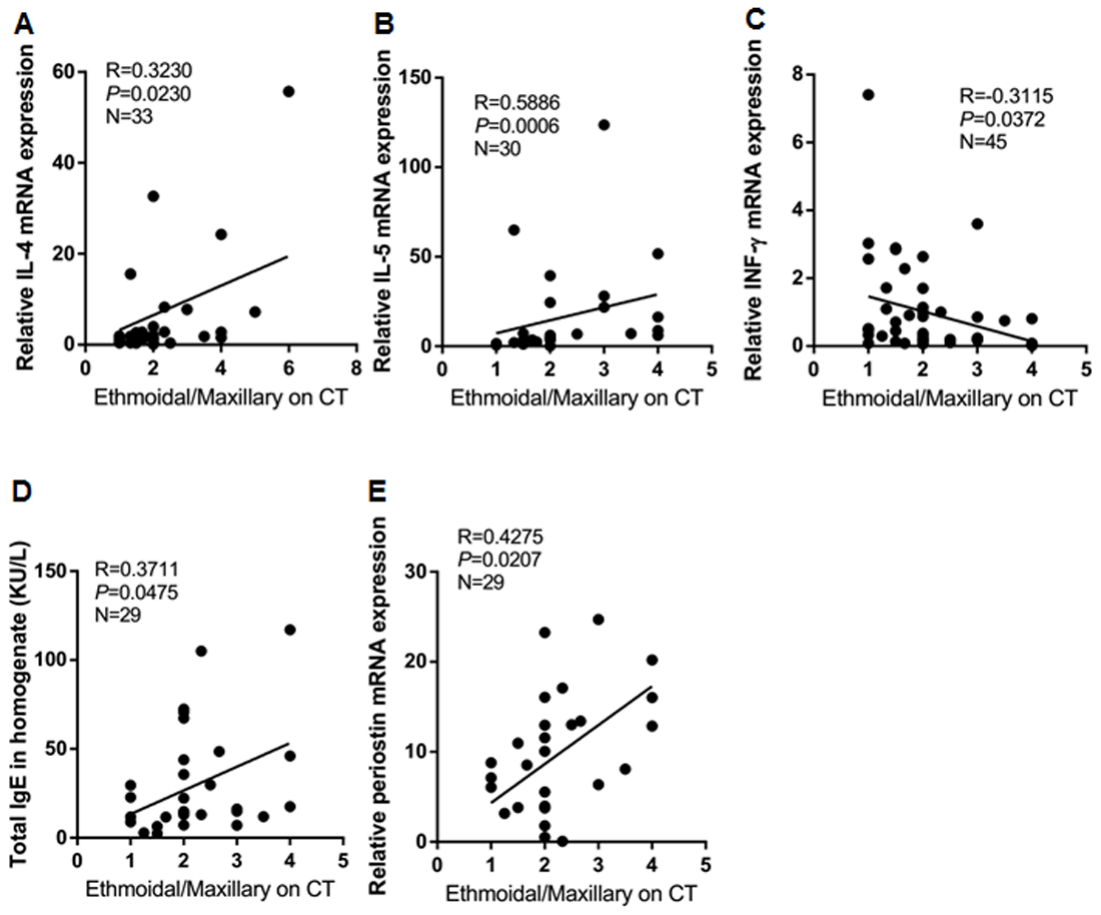
Correlation of the ratio of ethmoidal to maxillary CT scores with inflammatory markers in different endotypes of CRSwNP, including (A) IL–4 mRNA, (B) IL–5 mRNA, (C) IFN-γ mRNA, (D) total IgE homogenate, and (E) periostin mRNA.

To investigate whether or not difference in CT findings between NP subtypes is associated with various immunologic biomarkers, total IgE levels of nasal tissue homogenates and mRNA expression levels of IL–4, IL–5, IL–13, IFN-γ, TNF-α, IL-17A, IL–22, IL-23p19, TGF-β1, TGF-β2, TGF-β3, and periostin were assessed in CRSwNP patients (N = 62, Fig 4). We found that the E/M ratio on CT images was positively correlated with several inflammatory markers tested: IL–4 (r = 0.3230, *p* = 0.0230), IL–5 (r = 0.6358, *p*<0.0001), total IgE (r = 0.3711, *p* = 0.0475), and periostin (r = 0.4275, *p* = 0.0207), whereas it was inversely correlated with IFN-γ (r = -0.3115, *p* = 0.0357). However, there was no significant correlation with the subtraction of CT scores or olfactory opacification scores.

**Fig 5:**
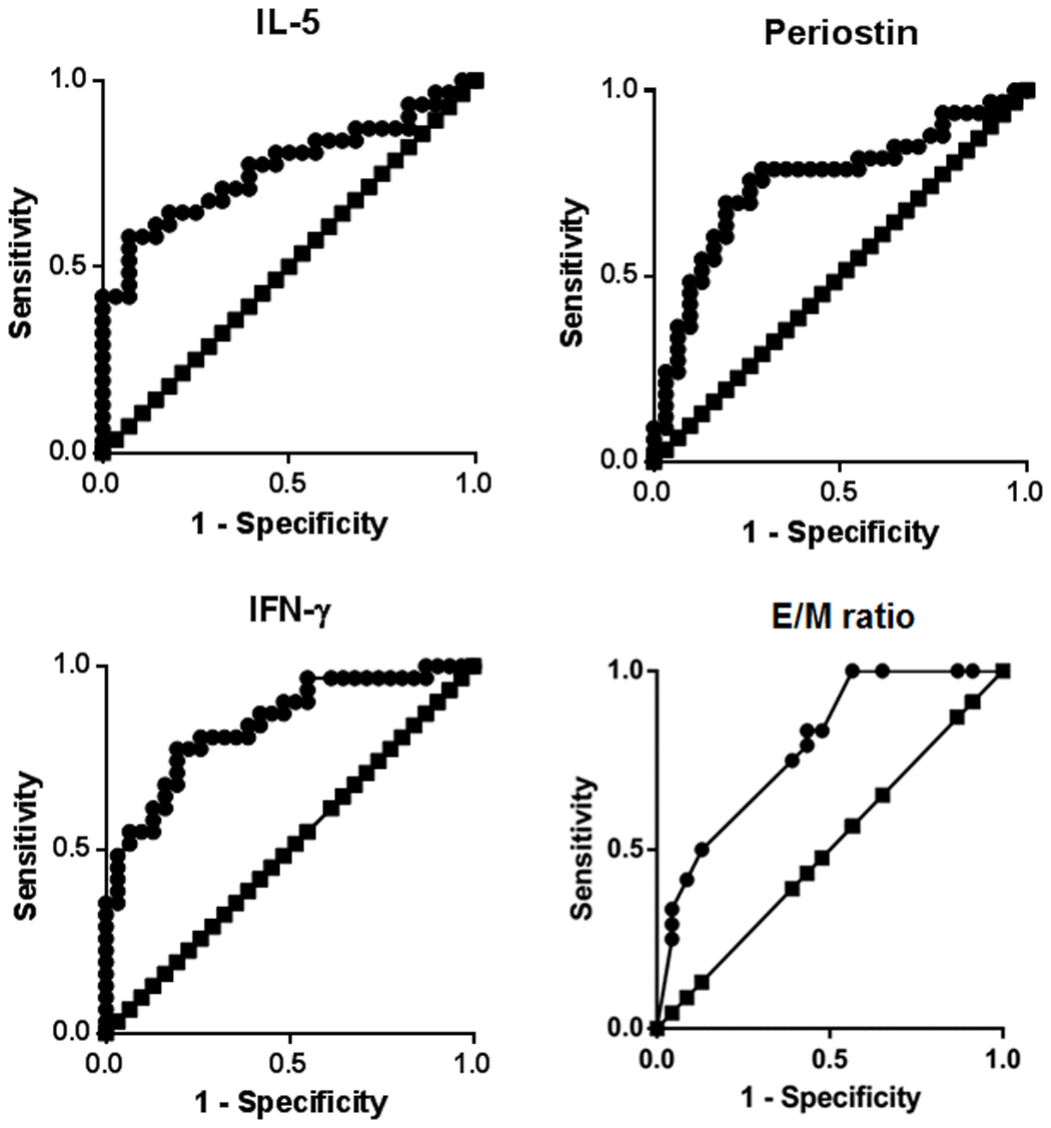
Receiver-operating characteristic curve analysis was used to identify the cutoff values for prediction of non-eosinophilic CRSwNP. The line with the squares is the result expected by chance. The line with the circles is the observed data. E/M ratio, the ratio of ethmoidal to maxillary CT scores.

Next line, we evaluated the diagnostic merits of non-eosinophilic CRSwNP by combination of important inflammatory markers and CT findings. In the ROC curve analyses, the cutoff of the IL–5, periostin, IFN-γ, and E/M ratio for patients with non-eosinophilic CRSwNP was <2.379 (sensitivity of 77.4%, specificity of 60.7%), <3.889 (sensitivity of 75.8%, specificity of 74.2%), >0.316 (sensitivity of 78.1%, specificity of 74.2%), and <2.167 (sensitivity of 75.0%, specificity of 60%) in the study population, respectively (Fig 5). The areas under the ROC curve for patients with non-eosinophilic CRSwNP were 0.7656 of IL–5, 0.7542 of periostin, 0.8251 of IFN-γ, and 0.7889 of E/M ratio, respectively in the study population. When it satisfies three of the four markers, we could suggest the non-eosinophilic CRSwNP with sensitivity of 84.4% and specificity of 84.8%.

## Discussion

Several studies have reported that more than one half of CRSwNP patients in Asia show non-eosinophilic inflammation [7–9, 15, 24, 26, 27]. The clinical and immunopathologic characteristics of non-eosinophilic CRSwNP are quite different from that of eosinophilic CRSwNP. For example, eosinophilic CRSwNP patients usually respond to steroid therapy, whereas non-eosinophilic CRSwNP patients are more likely to respond to macrolide treatment [28–31]. From an immunologic perspective, eosinophilic CRSwNP is associated with enhanced T_H_2 function and higher IL-5levels; whereas non-eosinophilic CRSwNP is associated with the combination of T_H_1/ T_H_2/ T_H_17 cell polarization [7, 8]. Thus, the heterogeneity of CRSwNP in Asian patients may lead to treatment difficulty. Distinguishing between eosinophilic and non-eosinophilic CRSwNP in Asian patients is necessary to provide the appropriate therapy and improve treatment outcome. In the present study, we found that infiltration of eosinophils was dominant in eosinophilic CRSwNP compared with non-eosinophilic CRSwNP, whereas neutrophil infiltration was increased in non-eosinophilic CRSwNP, which is consistent with outcomes from Asian studies [15, 24, 26].

Nasal and sinus epitheliums have important roles in the pathogenesis of CRSwNP [10]. Epithelial cell interacted with activated T cells which lead to induction of the proinflammatory function [32]. In addition, marked reductions in expression levels of several genes involved in epithelial barrier maintenance and repair occur in nasal polypogenesis [33]. Thus, we investigated the histological differences between eosinophilic and non-eosinophilic CRSwNP tissue, especially in the epithelium. Epithelial proliferation in the present study was determined by Ki-67-positive cell staining [34]. After Ki–67 immunohistochemical staining, we observed increased epithelial proliferation in non-eosinophilic NPs tissue. Moreover, pseudocyst formation, which is a form of epithelial alteration was detected at a low-power field (50×), primarily in non-eosinophilic NPs tissue. Pseudocyst also could be detected with endoscopic exam. Pseudocyst was visualized as a yellowish pocket inside of NP tissues upon endoscopic exam. To our knowledge, this is a novel finding concerning the differentiation between eosinophilic and non-eosinophilic CRSwNP, suggesting that endoscopy can be used to differentiate non-eosinophilic NP from eosinophilic NP. For example, pseudocysts were detected in only 1 out of 23 eosinophilic NP cases. Thus, the presence of pseudocysts in endoscopic exam likely rules out eosinophilic NP. Clinicians may apply this observation to subtype CRSwNP in an outpatient clinic setting without histological evaluation.

Interestingly, in our study, correlation analyses and inter-group comparisons of CT scores indicated that non-eosinophilic CRSwNP showed higher subtracted CT scores and lower E/M ratio in earlier disease extent, compared with eosinophilic CRSwNP. Meanwhile, the olfactory cleft opacification score were significantly lower in late stage of patients with non-eosinophilic CRSwNP than those with eosinophilic CRSwNP. These findings suggest that the prominent ethmoidal involvement and more diffuse inflammation were related with eosinophilic CRSwNP, while non-eosinophilic CRSwNP showed maxillary involvement and localized inflammation in early stage. In addition, we found that the E/M ratio was positively correlated with the expression of T_H_2 immune response (IL–4, IL–5, total IgE, and periostin) and negatively associated with the expression of T_H_1 immune response (IFN-γ). There are some studies for surrogate markers of CRSwNP subtypes, such as CT image parameters and peripheral blood count, have been reported [35–38]. However, previous surrogate markers did not show any correlation between those and immunologic parameters; thus, these markers did not reflect the immunopathologic features of CRSwNP. Therefore, our data supported that the combination of cutoff value on IL–5, periostin, IFN-γ, and E/M ratio could be one of useful surrogate markers for subtypes of patients with CRSwNP.

## Conclusions

Collectively, our results demonstrated increased epithelial proliferation and pseudocyst formation in patients with non-eosinophilic CRSwNP, compared with those with eosinophilic CRSwNP. Moreover, eosinophilic CRSwNP showed diffuse and bilateral sinus inflammation in the early stages of the disease and later propagated in ethmoid sinus and olfactory cleft. In contrast, non-eosinophilic CRSwNP was characterized by localized and unilateral sinus inflammation in the early stage and later progressed to a maxillary sinus-dominant pattern. In addition, the low E/M ratio shows a high degree of correlation with features of the immunologic background in non-eosinophilic NPs. Furthermore, the combination of cutoff value, including IL–5, periostin, IFN-γ, and E/M scores, may be considered the surrogate marker to determine patients with non-eosinophil CRSwNP.
